# Structural and Functional Properties of the Hepatitis C Virus p7 Viroporin

**DOI:** 10.3390/v7082826

**Published:** 2015-08-06

**Authors:** Vanesa Madan, Ralf Bartenschlager

**Affiliations:** 1 Department of Infectious Diseases, Molecular Virology, University of Heidelberg, 69120 Heidelberg, Germany; 2 Division of Virus-Associated Carcinogenesis, German Cancer Research Center (DKFZ), 69120 Heidelberg, Germany

**Keywords:** hepatitis C virus, p7 protein, viroporins, small membrane protein, ion channel activity, oligomeric structure, pore-like function, virus assembly and release, antiviral target

## Abstract

The high prevalence of hepatitis C virus (HCV) infection in the human population has triggered intensive research efforts that have led to the development of curative antiviral therapy. Moreover, HCV has become a role model to study fundamental principles that govern the replication cycle of a positive strand RNA virus. In fact, for most HCV proteins high-resolution X-ray and NMR (Nuclear Magnetic Resonance)-based structures have been established and profound insights into their biochemical and biological properties have been gained. One example is p7, a small hydrophobic protein that is dispensable for RNA replication, but crucial for the production and release of infectious HCV particles from infected cells. Owing to its ability to insert into membranes and assemble into homo-oligomeric complexes that function as minimalistic ion channels, HCV p7 is a member of the viroporin family. This review compiles the most recent findings related to the structure and dual pore/ion channel activity of p7 of different HCV genotypes. The alternative conformations and topologies proposed for HCV p7 in its monomeric and oligomeric state are described and discussed in detail. We also summarize the different roles p7 might play in the HCV replication cycle and highlight both the ion channel/pore-like function and the additional roles of p7 unrelated to its channel activity. Finally, we discuss possibilities to utilize viroporin inhibitors for antagonizing p7 ion channel/pore-like activity.

## 1. Introduction

**Figure 1:**
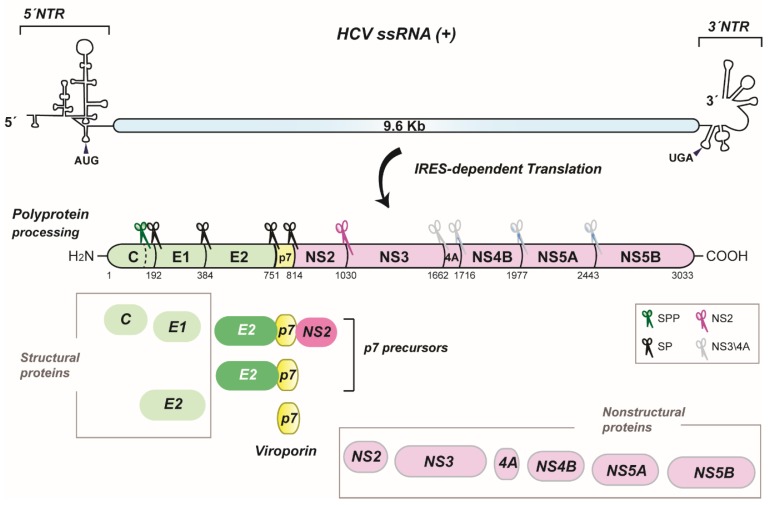
HCV genome organization, polyprotein processing and p7 precursors. A schematic representation of the HCV RNA genome including the non-translated regions (NTR) at the 5′ and 3′ ends and the coding region is shown on the top. AUG and UGA indicate the start and stop codons of the long open reading frame, respectively. The polyprotein precursor generated by IRES-dependent RNA translation and cleavage products are shown below. Numbers refer to amino acid positions of the JFH-1 isolate (GenBank accession number AB047639). Nonstructural proteins are depicted in pink color, p7 in yellow and structural proteins in green. Cellular and viral proteases responsible for polyprotein cleavage are represented by scissors and specified in the box. SPP, signal peptide peptidase; SP, signal peptidase. p7 precursors and p7 are highlighted.

The hepatitis C virus (HCV) forms the genus *Hepacivirus* within the *Flaviviridae* family. HCV is a positive-strand RNA virus encoding a single polyprotein precursor [1] that is generated by RNA translation at the rough endoplasmic reticulum (ER). This polyprotein is proteolytically processed into 10 mature proteins in a preferential, but not obligatory order (Figure 1) [2,3]. Of these, p7 separates the structural proteins (*i.e.*, Core and the envelope glycoproteins E1 and E2) from the nonstructural proteins that save for NS2 are essential for viral RNA replication (NS3, NS4A, NS4B, NS5A and NS5B) [4]. Importantly, polyprotein processing is a regulated event giving rise to cleavage intermediates, which is best illustrated with p7. In fact, the identification of p7 as an isolated product was not evident or predictable. This is due to inefficient cleavage of E2-p7-NS2 and E2-p7 precursors by signal peptidase at the ER [5,6,7] (Figure 1).

Initial attempts to ascribe distinct function(s) of p7 to the viral life cycle were complicated by the lack of adequate cell culture models to propagate HCV in cultured cells and limited availability of reliable antibodies recognizing p7. The initial development of a self-replicating subgenomic replicon, based on the HCV replicase factors (*i.e.*, NS3, NS4A, NS4B, NS5A, NS5B and the NTRs), demonstrated that the structural proteins and p7 were dispensable for viral RNA replication [8]. However, since these replicons only recapitulate the intracellular steps of the HCV replication cycle, the possible role of p7 for virus production could not be addressed. 

By that time it was speculated that p7 might be a membrane permeabilizing protein and involved in virus production/release [9]. This assumption was based on *in silico* prediction of p7 membrane topology that was similar to the p7 protein of the related pestiviruses and the 6K protein of alphaviruses, the latter being a well-studied member of the family of viroporins [10,11]. Strong support for the ion channel/pore-like activity of HCV p7 was obtained by *in vitro* studies demonstrating a partial selectivity for cations, but also for small molecules [12,13,14,15,16,17]. However, studies on the role of HCV p7 for virus production only became possible with the molecular cloning of a genotype (Gt) 2a isolate (designated JFH-1) from a Japanese patient suffering from fulminant hepatitis [18,19]. This isolate that efficiently replicates in human hepatoma cell lines and produces infectious virus particles was used to demonstrate that p7 is essential for particle assembly and release [20,21], a finding that is consistent with an earlier study demonstrating that p7 is essential for productive HCV propagation *in vivo* [22]. By using a series of intra- and intergenotypic chimeric HCV genomes, these observations were extended to many other HCV Gts [20,21]. Interestingly, by using HCV-like particles and p7 inhibitors a contribution of p7 as enhancer of virus entry has been proposed [23,24]. However, difficulties in detection of p7 in infectious virus particles, as well as the observation that deletion of p7 does not affect HCV specific infectivity raise controversies [20,25].

Viroporins are formed by a growing family of viral proteins identified in RNA and DNA viruses. These proteins are able to oligomerize, forming hydrophilic pores/ion channels in host cell membranes [26,27]. Viroporins share common features such as their small size, hydrophobicity, the presence of at least one amphiphatic transmembrane domain and the cytopathogenicity. The main consequence of viroporin expression during infection is the disruption of ionic gradients across target membranes thereby altering physiological cell functions [27,28]. On the other hand, viroporin action results in establishment of a favorable environment for different steps of the viral life cycle, very often, but not exclusively assembly of virions and their release from infected cells [26]. For instance, in case of HIV-1 Vpu, alphavirus 6K, influenza A virus M2, SARS-CoV (severe acute respiratory syndrome coronavirus) E and HCV p7, deletion of the viroporin coding sequence reduces the production of infectious virus particles and the pathogenicity [22,29,30,31,32,33].

In the following sections we will discuss the major findings related to the structure and function of HCV p7 viroporin, as well as its possible use for antiviral therapy.

## 2. HCV p7 Topology and Structure

**Figure 2:**
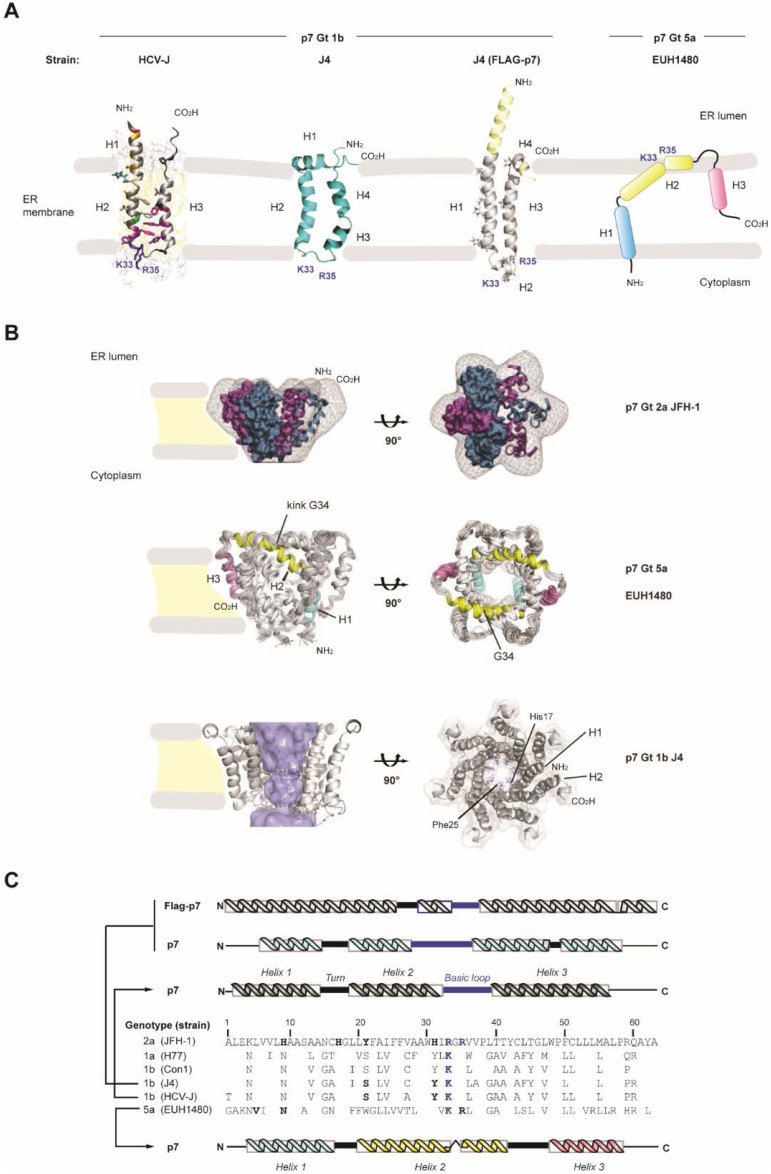
Structures of the p7 monomer and hexamer. (**A**) The membrane topology of HCV p7 Gt1b (HCV-J) as deduced from the NMR structure [17] is shown in the left. NMR-based structures of p7 (2MTS; middle. Adapted with permission from [40]. Copyright 2013 American Chemical Society) and Flag-p7 (3ZD0; right) of the Gt1b isolate J4 are shown as ribbons in the middle panels [39,40]. A schematic representation of the membrane topology of HCV p7 Gt5a as deduced from the NMR structure is shown in the right [35]. α-helices proposed for each structure are indicated. Basic conserved residues in the cytosolic loop of Gt1b p7 or in helix 2 of Gt5a p7 are indicated in blue bold letters. Yellow regions in the Flag-p7 structure represent the FLAG α-helix and extra residues. Note that shown monomeric structures of Gt1b p7 exhibit a “hairpin” conformation, whereas the p7 monomer of Gt5a adopts a “staple” conformation; (**B**) Upper panel: EM density map of the p7 hexamer of a Gt2a HCV isolate in vertical (left) and top (right) view [16]. Simulated p7 monomers (in purple and blue) were fitted into the flower-shape map with their N- and C-termini oriented to the petal tips. The topology of p7 is represented as described with both N- and C-termini exposed to the ER lumen [9,16]. Middle panel: NMR-based hexameric structure of p7 of a Gt5a HCV isolate (2M6X) [35]. α-helices in the monomeric structure are highlighted in colors. Residue Gly34 residing between the conserved basic residues, corresponds to a kink in the second α-helix. Bottom panel; heptameric model of p7 of the Gt1b HCV isolate J4 deduced from NMR data (3ZD0) [39]. The side chains of His17 and Phe25 are highlighted; (**C**) Multiple alignment of p7 sequences from representative Gts, including p7 of the two strains displayed in (**A**) and (**C**). Numbers refer to amino acid positions in p7 of Gt2a (JFH-1) which was used as a reference. Conserved basic residues are indicated in blue and bold. Residues highlighted in bold and black letters have been suggested to participate in ion gating and reside in the pore lumen of the oligomeric channel. Schematic representations of α-helical, turn and loop regions corresponding to the structures of the p7 monomers in (**A**) and (**B**) (same colors) are shown on the top and bottom, respectively.

Based on protein structure and membrane topology, two major classes (I and II) and two subclasses (a and b) of viroporins have been defined. A third class including viroporins with three hydrophobic domains has been also proposed [26]. In this respect, HCV p7 is a class IIA viroporin; it has a length of 63 amino acid residues forming two transmembrane spanning regions that are separated by a basic cytoplasmic loop with both N- and C-termini facing the ER lumen [9] (Figure 2A). However, an alternative topology with the C-terminus exposed to the cytoplasm has been proposed as deduced from the expression of an E2-p7 precursor protein [34]. The tendency of p7 to form homo-oligomers and to assemble into ion channel-like structures *in vitro* has been reported by several laboratories [12,13,14,15,16,17,35]. These results are based on the use of recombinant p7 or synthetic peptides inserted into artificial lipid bilayers, overexpression of p7 in Xenopus oocytes and electrophysiological recording techniques, including patch-clamp and two-electrode voltage clamp [12,13,14,15,17,35]. Supported by electron microscopy (EM), biochemical data and computational modeling techniques, *in silico* analysis of the HCV p7 sequence predicted a hairpin-like structure of the monomer with the N-terminal transmembrane domain (TMD) lining the lumen of the pore [12,36,37] (Figure 2A). Initial studies using transmission EM (TEM) and computer-based image analysis revealed that p7 fusion proteins form a mix of hexameric and heptameric complexes in liposomes [12,15]. Results obtained with equilibrium sedimentation experiments of p7 were consistent with the formation of different p7 oligomeric states comprising 6 ± 1 subunits, with the heterogeneity depending strongly on detergent [17]. Thus, in biological membranes p7 might exist as a mixture of oligomers with variable size. A subsequent TEM study combined with single particle reconstruction of chemically synthesized full-length p7 of Gt2a, solubilized in short chain DHPC (1,2-diheptanoyl-snglycero-3-phosphocholine) detergent micelles at pH 7.0, provided the first 3D structure of hexameric p7 channels at a resolution of 16Å [16]. It was found that the p7 channel exists as a flower-shaped complex with 6 petals emerging from a conical base. The generation of the first EM density map from this study allowed modeling of simulated p7 monomers into the hexameric volume with a high fitting value (97.3%) as well as to predict interactions between them at the lower part of the channel. Moreover, a topology of the complex indicating that the N- and C-termini in each monomer are exposed towards the tip of the petals could be confirmed by immunogold labeling with p7 specific antibodies. Accordingly, the more open part of the channel was suggested to protrude into the ER lumen (Figure 2B). This EM-based 3D model was subsequently adopted as a useful template to reconstruct p7 channels of other Gts from NMR-solved monomeric structures [38].

Currently, four NMR-based structures of the HCV p7 monomer have been reported (Figure 2A). First, Monserret and coworkers obtained the structure of the second TMD of p7 from Gt1b dissolved in trifluoroethanol/water mixture and presented a model of the p7 monomer based on structural data from experiments in membrane mimetics [17]. A NMR-structure of full-length p7 was obtained in methanol by Foster and colleagues [39]. The structure by Cook and coworkers revealed Gt1b p7 monomers at pH 4.0 and reconstituted into micelles formed by DHPC containing short acyl chains [40]. Interestingly, the NMR-based structure reported by Ouyang and coworkers was determined with p7 in micelles of DPC (*i.e.*, dodecylphosphocholine containing longer acyl chains) and was assumed to have hexameric oligomeric size on the basis of parallel negative staining EM experiments [35].

While clear similarities were found between different subtypes of HCV p7 from Gt1b, notable discrepancies were apparent to the structure of the Gt5a p7 [35] (Figure 2A,B). Whereas the monomers of Gt1b exhibit a classic “hairpin-like” conformation, Gt5a p7 monomers display an extended and more complex distribution of α-helical elements tilted in an unusual way (Figure 2A,B). In the hairpin model, three or four helical segments can be distinguished with variable positioning depending on the reported structure (Figure 2C). In the structures by the groups of Opella and Penin, an N-terminal α-helix connected to the first TM helix by a flexible turn has been proposed to interact with the head groups of the lipid bilayer as deduced from molecular dynamics simulations [17,40]. This helical region at the membrane interface might adopt an almost perpendicular angle to the subsequent transmembrane helix [40]. In the structure by the Griffin group these first two helical segments of a Gt1b p7 (isolate J4) appear as a single transmembrane helix [39] (Figure 2A). In addition, in all Gt1b structures a cytosolic loop separates the first transmembrane region from the second one, the latter exhibiting certain variability and including one or two helical elements at different positions [17,39,40]. The most C-terminal part has been proposed to be either structured or to include a short and flexible helix (Figure 2A,C). Overall, p7 appears to be a flexible protein as supported by the structural differences observed by various groups. Very important to note is the role of lipids or organic solvents that define the composition and thickness of the membrane thus influencing the final conformation of p7 [37,41].

Despite the differences in number and distribution of secondary structure elements within Gt1b p7 monomers, it is surprising that all of these channel subunits share a hairpin conformation with anti-parallel TM regions slightly tilted to the lipid bilayer (Figure 2A,C). In this regard, it is unclear why the p7 monomer from Gt5a presents an unusual and distinct conformation (Figure 2A, right panel). Nevertheless, this structure represents the first example in which the conformation of p7 subunits was extracted from a NMR-based hexameric complex [35]. It reveals three alpha helical regions tilted with sharp angles and adopting a curved “staple-like” conformation where the first and third helix are separated through a second helix kinked at Gly 34 (Figure 2B). Interestingly, this residue is located between two highly conserved basic amino acid residues (K/R33, R35) that are crucial for virus particle infectivity *in vivo* and important for p7 ion channel activity [22,42]. This different positioning of the helical elements between p7 proteins of different Gts and a more extended arrangement of the monomer within the assembled channel revealed unexpected features when compared with models of Gt1b p7 such as the lack of an unstructured loop connecting the two transmembrane regions or a C-terminal region that is embedded into the lipid bilayer (Figure 2B). 

The study by Ouyang and coworkers also provided important details on the overall architecture of the pore. Interestingly, the p7 oligomer embedded into the membrane exhibits a funnel-like shape resembling the flower-like shape model and fitting within its EM map with high accuracy (correlation coefficient 0.94), even though the detergent concentration used to reconstitute p7 for NMR and EM analysis was not the same. This hexameric architecture is stabilized by multiple interactions amongst the monomers in such a way that the third C-terminal helix (H3) of one monomer interacts both with the second helix (H2) of the neighboring monomer and with the first N-terminal helix (H1) of the consecutive one (Figure 2B). This complex rearrangement of subunits might evoke a process of co-folding, at least under the *in vitro* conditions used during protein “renaturation” in the presence of DPC [43]. However, it remains enigmatic whether such a structure could be obtained in a physiological context since such co-folding cannot be explained by our current understanding of protein folding *in cellulo*. The fact that intra-monomer interactions, suggested from previous NMR- and EM-based studies, are absent in this structure is novel and noteworthy. In this NMR structure, the narrower part of the hydrophilic channel is formed by tight association of H1 helices (N-termini), followed by a wider region formed by H2 helices. According to this study the structural protrusions or “petals” mainly correspond to the H3 helices surrounding and packaging the central cavity while the second half of H2 helices and H2-H3 interconnecting regions represent the petals tips. Therefore, this flower-like architecture presents a topology distinct to that previously described [16,35] (Figure 2B). The existence of alternative structures between p7 monomers from Gt1b and Gt5a could be explained, at least in part, by the genetic diversity of the amino acid sequences and by used experimental conditions employed in each study. However, other possibilities might account for apparent structural discrepancies linked to Gt. Further characterization of the p7 structure ideally in cells infected with HCV of different Gts will help to corroborate this new finding. 

Models for hexameric or heptameric p7 channels from Gt1a, 1b and 2a have been obtained by molecular dynamics using either predicted or NMR-based structures of the corresponding monomers inserted into lipid bilayers [37,38,39,40]. In these studies p7 bundles display a luminal orientation of the first TMD with the C-terminal TM region facing the membrane hydrophobic phase and surrounding the channel (Figure 2B). Strikingly, *in silico*-based studies of the secondary structure of the Gt5a p7 monomer predicts a hairpin-like fold reminiscent of the NMR structure of Gt1b p7 [38]. On this basis, the reported curved “staple-like” conformation of the Gt5a monomer assembled into the channel could not have been predicted *a priori*. Similarly, the structure of the complete Gt5a oligomeric p7 complex reported by Ouyang and coworkers is not expected in the light of the computer-predicted conformation of the p7 monomer. Thus, one can expect that conformational changes are induced in the monomeric p7 hairpins upon oligomerization. In this case p7 monomers might exert different functions as compared to oligomers, which is consistent with the different reported functions of p7 in the HCV life cycle that are either dependent or independent on the ion channel activity (see sections below). However, other analyses of the Gt5a p7 hexamer revealed features arguing against the existence of such a structure in a physiological membrane environment. One of them is the presence of 18 positively charged side chains corresponding to arginine residues exposed to the hydrophobic membrane phase [44]. Although this might be due to micelle composition and shape, it is difficult to reconcile in a cellular membrane environment. Another example is the existence, in p7 hexamer, of several hydrophilic cavities embedded into the hydrophobic core of the lipid membrane [41]. Thus, in view of the very flexible structure of p7 and its interaction with lipids, obtaining a physiologically relevant structure will require the study of p7 in a true membrane-like environment mimicking composition and thickness of the ER.

Although it is well established that p7 of different HCV Gts forms homo-oligomers of heterogeneous size *in vitro*, the existence of such a mixture of p7 species in infected hepatocytes remains to be determined. Additionally, initial studies of p7 membrane topology in cells were based on the expression of isolated p7 or the E2-p7 precursor and, therefore, validation in infection-based systems using full-length HCV genomes encoding fully functional (tagged versions) of p7 from different Gts will be required. The biochemical characterization of p7 complexes forming in infected cells should allow to determine the physiological relevance of the structures/conformations proposed for the p7 monomer that, so far, are predominantly deduced from NMR-based approaches *in vitro*.

## 3. HCV p7 Ion Channel and Pore-Like Function

Due to its small size, high hydrophobicity, and its tendency to form oligomers in cellular membranes, viroporins are ideal candidates to explain changes in membrane permeability occurring in cells infected by different viruses [26]. This membrane alteration has been further described in studies assessing the entry or release of small compounds into and out of cultured cells and artificial liposomes. For instance, the antibiotic hygromycin B (HB; molecular weight of 527.5 Dalton) has been extensively used as a probe to evaluate the increase of membrane permeability upon individual expression of various viroporins [45,46,47,48,49,50,51,52,53,54]. Although this is an indirect assay (*i.e.*, HB selectively enters cells with enhanced membrane permeability and inhibits protein synthesis) and the presence of viroporins at the cell surface is not a common hallmark of virus-infected cells, this approach has been invaluable to study the viroporin function of many family members. Nevertheless, whether the entry of HB into cells occurs directly via viroporin-assembled pores at the cell surface or indirectly by upregulation of endocytosis remains to be explored. Other biophysical assays monitoring the release of dyes with defined size from preloaded liposomes were initially used to establish the pore-like properties of these proteins as well as to predict the size of their inner channel [55,56,57]. Similar liposome-based approaches using small fluorescent probes such as carboxyfluorescein (0.37 KDa) or the pH-sensor HPTS (8-hydroxypyrene-1,3,6-trisulfonic acid; 0.52KDa) were employed to corroborate the formation of pores by HCV p7 of different Gts [24,58]. This system has also been adapted for high throughput screenings aimed at finding HCV p7-specific inhibitors [40,59,60]. Taken together, p7 non-selective pore properties are evidenced from all these *in vitro* assays, however further studies are required to corroborate the assumed direct passage of large molecules into cultured cells via p7.

While the M2 protein of influenza A virus and the Kcv protein of chlorella virus PBCV-1 are highly selective for proton and potassium, respectively [61,62], most viroporins show low ion selectivity. This appears to be the case also for HCV p7 that has minor preference for cations *vs.* anions as shown by electrophysiological recording techniques in artificial bilayers and *Xenopus laevis* oocytes [12,13,14,15,17,35]. Recent studies provided strong evidence for proton conductance of p7 from different Gts (*i.e.*, 1a, 1b and 2a) in isolated intracellular membrane vesicles and in HCV-infected cells [58,63].

The functional resemblance of HCV p7 with the M2 viroporin of influenza A virus (IAV) and their common acid-activated proton channel activity suggests that both viroporins might share a similar gating mechanism for protons. The gating mechanism of M2 has been extensively studied. It involves a sequence motif in the TM region with two key amino acids that project to the center of the pore: His37, the proton-conducting residue, and Trp41, identified as the proton “gate” [64]. Based on NMR data obtained with monomeric p7, potentially equivalent residues line the lumen of the channel. These residues correspond to conserved amino acid residues His17 and Tyr/Trp 21 present in Gt2a and 5a, but absent in Gt1a or 1b, where Ser is found (Figure 2C). The functional relevance of these and other residues that differ between the Gts was confirmed in several mutagenesis-based studies [20,65,66,67,68,69,70]. Moreover, a role for Phe25 as “gate” residue has been suggested from liposome-based assays measuring the enhanced permeabilization capacity conferred by an alanine substitution for Phe25 [71]. By using molecular dynamics simulations this Phe25 residue, together with Ile32, was observed to form an energetic barrier to water permeation [37]. Consistent with its sequence and structural divergence, the hexameric structure of Gt5a p7 revealed an alternative gating mechanism on the basis of its hypothetical cation selectivity. Here, two constriction points would regulate the ion flux: a basic ring formed by Arg35 at the wider (C-terminal) opening and a double ring including Asn9 and Ile6 at the narrow base. Although cation channel activity has not been proven for Gt5a p7, in the case of Gt2a, mutations of amino acids at equivalent positions (Arg35; His9) decreased cation channel activity [35]. Whilst the structural data is not conclusive to validate the proposed gating system for Gt5a, it still supports the functional influence of these residues on the ion channel activity of p7. Additional channel structures of p7 will be required to elucidate whether Phe25 and Ile32 of p7 function as gate residues in all HCV Gts or just in some distinct Gts or subtypes. Moreover, it has to be taken into account that the lipidic environment influences p7 structure and, thus, its electrophysiological properties [72]. In fact, a recent study suggested that two different conformations of p7 of Gt1a, presumably influenced by the lipidic environment, likely account for proton selectivity (influenced by the content of α-helix structure) and permeability to large molecules (affected by the content of β-structures), at least in liposome-based assays [73]. Even though the high content of β-structure is thought to be an artifact, this study corroborated the plasticity of the p7 structure and suggests that alternative conformers of p7 might exist thereby explaining the multiple functions of p7 in HCV infected cells.

Although great advances have been made in the HCV field in the last few years, we are still far away from a comprehensive understanding of the structure–function relationship of the p7 viroporin. This is mainly due to use of different HCV Gts and subtypes, the presence of tags used for protein purification and detection, as well as experimental conditions and technologies, applied in different laboratories. Thus, future research in this field will require multidisciplinary and Gt cross-comparative analyses of p7 to discern which of the reported biophysical and functional *in vitro* channel properties are acting in HCV-infected cells.

## 4. Role of p7 Channel Activity in Production and Release of Infectious HCV Particles

It has been proposed that p7 acts as a low pH-activated channel in HCV-infected cells, similar to the IAV M2 viroporin, by equilibrating the proton gradient across the membrane of acidic compartments [58,63,64]. The dissipation of protons from the lumen of acidic compartments to the cytoplasm results in alcalinization of such organelles that is required for final steps of virus assembly and subsequent egress of virions [20,58,63]. Mutants in which the conserved dibasic motif in the cytosolic loop is affected are unable to regulate the proton gradient, resulting in impaired pore-like and ion channel activity in artificial lipid bilayers [12,58,63]. Moreover, peptides containing such mutations show reduced membrane binding affinity [74]. Although some of these mutations are associated with delayed cleavage of the E2-p7-NS2 precursor (e.g., RR33/35 in Gt2a and KR33/35 in Gt1b and 1a) or E2 degradation (e.g., RGR33-35/AAA), which might account for impaired virus production, recent studies have now confirmed the importance of the basic residues for p7 ion channel activity and point to a direct link between the proton channel activity of p7 and production of HCV infectious particles [20,33,63]. Consistent with this observation is the partial restoration of virus production by the IAV M2 viroporin or the rescue of a Gt2a p7 mutant by Gt1b p7 using a transcomplementation assay [58,63]. Likewise, a partial rescue of infectious virus production was achieved by pharmacological inhibition of endosomal vesicle acidification using bafilomycin A1, an inhibitor of the vacuolar-type proton-ATPase. Accordingly, a pH-dependent maturation process has been suggested to precede the secretion of infectious virions. This assumption is reinforced by the fact that infectious intracellular particles are highly sensitive to treatment with acidic pH whereas secreted virions are acid-resistant [58]. Moreover, the acid stability of secreted virus particles seems to be influenced by p7 in a Gt-dependent manner [69]. Considering all these data, p7 ion channel might be regarded as a modulator of the pH-mediated maturation process of HCV particles. However, how this maturation process is regulated and confers the “acid resistance” signature to secreted particles during exit is not known. Prevention of premature conformational changes in the HCV envelope glycoproteins, reminiscent to the function of IAV M2 as proposed by others is an attractive possibility that deserves further investigation [58,75]. It has recently been shown that the HCV envelope glycoproteins interact with apolipoproteins which are an integral component of infectious virions [76,77,78,79]. Since p7 also interacts with the envelope glycoproteins, it is tempting to speculate that p7 might also influence apolipoprotein incorporation into HCV particles or particle maturation, thus contributing to virus production and infectivity.

## 5. Functions of p7 Unrelated to the Ion-/Proton Channel

Biochemical studies have confirmed that p7 mainly, but not exclusively, interacts with NS2, which is assumed to act as an organizer of virion assembly [25,80,81,82,83,84,85]. This interaction does not require the p7 ion channel activity, but instead likely serves to recruit the envelope glycoproteins, via p7 interaction, which in turn associates with NS2 to the sites of HCV assembly. In this way p7 can regulate the subcellular localization of NS2 and core protein [85,86]. Additionally, p7/NS2 association has been shown to dictate the relocalization of core from LDs to ER in infected cells. This redistribution, likely required for HCV assembly and reflecting assembly efficiency, correlated with high infectivity titers [86]. Apart from these p7 interactions, a recent study of the interaction network of HCV proteins has pointed to core and the two envelope glycoproteins E1 and E2 as new direct binding partners of p7 [87], while genetic data suggest an NS2-dependent association of p7 with NS5A [88].

By using expression-based systems, p7 was found to localize mainly at the ER, but also in membranes close to mitochondria and at the plasma membrane [9,58,89,90]. However, these studies have been limited because of the lack of p7-specific antibodies and therefore, tagged versions of p7 with unclear physiological functionality had to be used. Only recently, several groups have generated JFH1-derived full-length HCV genomes encoding double HA-tagged or chimeric 2a/1b p7 proteins that are fully functional. These studies revealed a predominant localization of p7 at the ER and the perinuclear region. Moreover, p7 was found to colocalize with the E2 glycoprotein as well as NS5A, core, NS2 and NS3 [25,63].

It is generally assumed that nucleocapsid formation and envelopment of HCV particles are coupled reactions. Thus, a block in envelopment would prevent nucleocapsid assembly, concomitant with an intracellular accumulation of core protein on the surface of LDs and of the envelope glycoproteins in the vicinity of assembly sites. Consistent with these assumptions, studies of p7 dibasic motif mutants revealed a Gt-dependent reduction of intracellular infectivity, likely resulting from the incomplete incorporation of viral RNA into nucleocapsids that aberrantly accumulate on the surface of LDs, as demonstrated with the Jc1 (J6/JFH1) HCV chimera [63,91,92,93]. Moreover, these immature capsids lack the ER membrane-derived viral envelope. Strikingly, non-infectious nucleocapsid-containing particles with density comparable to infectious HCV, and thus likely possessing an envelope with E1/E2 glycoproteins, were isolated from cells infected with mutant HCV of the JFH-1 isolate. These apparent discrepancies between viruses with different assembly fitness could be explained, at least in part, by Gt-specific interactions between p7, NS2 and the structural proteins [86].

Overall these findings suggest that p7 is involved in consecutive steps during assembly of HCV particles including the early encapsidation of the viral genome, the relocalization of RNA-containing core complexes from LDs to the ER and eventually the acquisition of the membrane envelope. The fact that these steps are not sufficient to confer infectivity to viral particles of the JFH-1 isolate, argues that p7-mediated particle maturation is a late and rate-limiting step for production and release of infectious HCV virions. In this respect, p7 would have two functions: first, to participate in the envelopment of virus particles by mediating the interaction between the envelope glycoproteins and NS2 (which in turn via association with NS3 “recruits” the replicase) and second, by mediating or regulating an intracellular maturation step of HCV particles to render them pH-resistant and fully infectious.

## 6. HCV p7 Channel as Antiviral Drug Target

A number of compounds have been reported to exert an inhibitory effect on HCV p7 channel activity. These comprise classic inhibitors of the IAV M2 viroporin, such as the adamantanes, amantadine and rimantadine, iminosugar derivatives (e.g., *N*-nonyl deoxynojirimycin,*N*N-DNJ), hexamethylamiloride (HMA) and GSK-2, a specific compound developed by Glaxo-Smith-Kline. Their effect on ion channel/pore-like activity of p7 *in vitro* has been assessed either by electrophysiological recording techniques using p7 reconstituted in artificial lipid bilayers or by liposome-based assays [13,14,39,58,59,68,94]. Overall, these compounds inhibit p7 depending on the HCV Gt and subtype and with various efficiencies. For instance, higher theoretical binding affinity of rimantadine to p7 of Gt1b and Gt2a (Kd 0.251 and 0.617, respectively) as compared to amantadine (Kd 1.32 and 7.41, respectively) is reflected by its lower Kd values and explains its increased inhibitory potency [71].

Several of these compounds have also been studied in infected cells and were found to reduce virus production up to 10-fold in a Gt-dependent manner [20,39,95]. This inhibitory phenotype is characterized by a reduced secretion of HCV particles without impairment of infectivity of intracellular virions [20,24,71]. For most of these compounds, the mechanism responsible for the reduction of virus secretion is poorly understood. In case of the iminosugar derivative *N*N-DNJ, inhibition of α-glucosidases, leading to altered glycosylation pattern and total level of HCV E2, might be a mechanism adding to the direct binding and inhibition of p7 [13,24]. However, it has recently been reported that productive HCV release is linked to inhibition of acidification of virus-containing compartments mediated by p7 and this process is blocked by adamantanes and HMA [58].

Consistent with the proposed role of the p7 ion channel for the early infection steps of HCV, a Gt-dependent block of virus entry has been described for adamantanes, GSK-2 and *N*N-DNJ [24]. However, the observation that an inactive p7 mutant does not affect specific infectivity of released HCV particles is not consistent with such a mechanism of p7 inhibition [20]. Moreover, the involvement of p7 ion channel activity in cell-to-cell transmission of HCV is unlikely as deduced from the limited inhibitory effect of p7 antagonists on this mode of virus spread [24,96]. Thus, p7 is primarily considered an attractive target to block virus release.

Taking advantage of the 3D structure of the p7 heptamer (Gt1bJ4), in silico-based screening of potential p7 ligands has been conducted leading to the identification of a series of compounds with improved inhibitory effect on the J4/JFH-1 virus chimera [39]. These compounds bind to a peripheral allosteric site in p7 predicted to be the rimantadine binding pocket. Consistently, secretion of a rimantadine-resistant virus containing a phenylalanine substitution for Leu20 in p7 is suppressed by these new drugs. Additional encouraging data show that doses at the compound concentrations in the low micro molar range are sufficient to suppress the production of chimeric viruses derived from several non-1b Gts. Thus far, resistance against these novel p7 inhibitors has not been reported.

Clinical development of p7 inhibitors has met limited success. Consistent with the very low antiviral efficacy of amantadine in HCV cell culture systems [20], no clinical benefit of amantadine-based combination therapy was found in comparison to standard of care (peg-IFNα plus ribavirin) [97]. Thus far, the only promising effect reported for a triple therapy of amantadine/pegIFNα/ribavirin was an early reduction of viremia, but this effect was not accompanied by a sustained virological response [98]. Perhaps the most efficient effect for a p7 inhibitor has been reported for BIT225, an amiloride derivative developed by Biotron that targets in addition to HCV p7 the HIV viroporin Vpu and p7 of the pestivirus BVDV both *in vitro* and in infected cells [99,100]. The inhibitory effect of BIT225 on the HCV p7 channel has been suggested by docking assays and confirmed in lipid bilayer systems, although no data have been reported in the context of HCV infection [100,101]. These docking studies revealed that amiloride derivatives seem to bind best to hexameric p7 Gt1a, with BIT225 exhibiting the lowest binding energy (−25.5 Kcal/mol) as compared to other p7 inhibitors such as amantadine (−7.9 Kcal/mol) and *N*N-DNJ (−6.5 Kcal/mol) [101]. One early clinical trial (phase Ib/IIa) in 24 treatment naïve Gt1 HCV patients indicated a significant reduction of viral load after twelve weeks treatment with BIT225 in combination with IFNα/ribavirin (www.biotron.com.au: biotron 2011).

Currently, a phase 2a/2 clinical trial is in progress and first results indicate activity against several HCV Gts, including the difficult-to-treat Gt3a and 1a (www.biotron.com.au; 29 May 2015). Moreover, BIT225 might be suitable for treatment of HCV/HIV co-infected patients. However, in the light of the high potency of available inhibitors targeting the NS3 serine-type protease, the NS5A replicase factor and the NS5B RNA-dependent RNA polymerase, the clinical niche of p7 inhibitors is unclear.

One limitation of selective antiviral therapy is drug resistance and unfortunately, p7 inhibitors are not an exception. In the case of adamantanes, the Leu20Phe mutation in p7 conferring rimantadine resistance was found in patients not responding to the triple therapy [71]. In case of the iminosugar derivatives that disrupt p7 oligomerization and thus, channel activity, a Phe25Ala polymorphism present in HCV Gt 3a but not in Gt1b was shown to mediate *N*N-DNJ resistance by stabilization of the p7 channel [71].

## 7. Conclusions

With the availability of efficient and fully permissive cell culture models for HCV, our knowledge about the role of p7 in the viral life cycle has increased profoundly. It became clear that this protein plays a major role in the assembly and release of infectious HCV particles. This appears to be mediated by two complementary functions of p7. First, by associating with the envelope glycoproteins and NS2 it participates in the coordination of the envelopment of virus particles and second, by forming a channel, it appears to be required for an intracellular maturation step of virions required for their secretion and rendering them pH resistant. The recent resolution of the p7 homo-hexamer structure by means of cryo-EM and NMR revealed an unexpected arrangement of p7 subunits that is different from the typical hairpin conformation and that proposes a novel gating mechanism. While these are notable achievements, important questions still remain to be solved. For instance, what is the basis for the structural diversity of the p7 ion channel? How does p7 contribute to virus release? How does p7 render intracellular HCV particles pH resistant? While from a clinical point of view, chronic hepatitis C might be regarded as a solved problem, studies of HCV will continue to provide important insights into the intricate virus–host cell interplay. In this respect, studies of HCV p7 will help to unravel how viroporins that are also used by many other viruses promote the production of infectious progeny particles and eventually contribute to pathogenicity.
